# Case Report: The cystic masquerade in type 1 Von Hippel-Lindau disease: coexisting clear cell renal cell carcinoma and an imaging-suspected pancreatic neuroendocrine tumor

**DOI:** 10.3389/fonc.2026.1922111

**Published:** 2026-09-14

**Authors:** Daiyu Yang, Yinghao Sun, Shengyu Zhang

**Affiliations:** Department of Gastroenterology, Peking Union Medical College Hospital, Peking Union Medical College and Chinese Academy of Medical Sciences, Beijing, China

**Keywords:** case report, obstructive jaundice, pancreatic neuroendocrine tumor, renal cell carcinoma, retinal hemangioblastoma, VHL variant, von Hippel-Lindau disease

## Abstract

VHL disease is a rare autosomal dominant tumor predisposition syndrome characterized by the development of cystic and solid lesions in multiple organs. Pancreatic and renal cysts are common manifestations and are often considered benign; however, clinically significant neoplasms may arise within or adjacent to these lesions, creating diagnostic challenges. We report a 54-year-old man who presented with progressive obstructive jaundice. Imaging revealed multiple pancreatic cysts, a cystic-solid lesion in the pancreatic head, a hypervascular pancreatic neck nodule suspicious for pNET, a giant left renal mass, bilateral renal cysts, and a Bosniak IV cystic lesion in the right kidney. His medical history was notable for retinal hemangioblastoma resulting in blindness of the right eye more than 30 years earlier. Left radical nephrectomy was performed, and pathology confirmed ccRCC. The pancreatic lesion remained an imaging-suspected pNET rather than a pathologically confirmed neoplasm. Germline testing identified a likely pathogenic VHL variant, confirming the molecular diagnosis of VHL syndrome. Family screening revealed that one of the patient’s two sons carried the same variant. The patient subsequently received belzutifan therapy from December 2025 to March 2026. Follow-up contrast-enhanced CT showed no progression of the residual lesions, consistent with short-term radiographic disease stability. This case highlights that extensive cystic disease in VHL syndrome may complicate the recognition and characterization of coexisting solid neoplastic lesions. Particular attention should be paid to enhancing mural nodules, hypervascular solid components, and interval growth when evaluating extensive cystic disease, as these findings may indicate coexisting clinically significant neoplastic lesions requiring further evaluation. Early recognition of VHL syndrome and timely genetic evaluation are essential for preventing delayed diagnosis and organ-threatening complications.

## Introduction

Von Hippel-Lindau (VHL) disease is a rare autosomal dominant hereditary syndrome caused by germline mutations in the VHL gene located on chromosome 3p25-26, with an estimated incidence of approximately 1 in 36,000 (1, 2). The disease is characterized by the development of a broad spectrum of tumors and cysts affecting multiple organs (3, 4). Loss of VHL protein function leads to accumulation of hypoxia-inducible factors (5), which in turn activates downstream pathways, including vascular endothelial growth factor (VEGF), platelet-derived growth factor (PDGF), and erythropoietin (EPO), thereby promoting tumorigenesis and angiogenesis across various tissues (6–8).

Clinically, VHL syndrome is classified into distinct subtypes based on genotype-phenotype correlations: type 1 (absence of pheochromocytoma), type 2A (presence of pheochromocytoma without renal cell carcinoma), type 2B (presence of both pheochromocytoma and renal cell carcinoma), and type 2C (isolated pheochromocytoma) (9, 10). These subtypes reflect the heterogeneity of disease manifestations and underline the importance of individualized clinical surveillance. VHL-associated lesions involve multiple organs, include retinal and central nervous system (CNS) hemangioblastomas, Endolymphatic sac tumors, renal cell carcinomas or cysts, pheochromocytomas, Epididymal cystadenoma, as well as pancreatic cysts and pancreatic neuroendocrine tumor (pNET) (8).

Pancreatic involvement is observed in up to 70% of patients with VHL disease and most commonly manifests as simple cysts or serous cystadenomas (11). Renal manifestations include both renal cysts and clear cell renal cell carcinoma (ccRCC), which remains the leading cause of disease-related mortality. Although pancreatic and renal cystic lesions are generally considered benign, clinically significant solid neoplasms may coexist with cystic disease, creating diagnostic challenges and potentially delaying recognition of lesions requiring intervention.

Here, we report a genetically confirmed case of type 1 VHL disease presenting with obstructive jaundice and extensive pancreatic and renal cystic disease coexisting with a pathologically confirmed ccRCC and an imaging-suspected pancreatic neuroendocrine tumor. This case highlights how widespread cystic disease may complicate the recognition and characterization of coexisting solid neoplastic lesions, creating a “cystic masquerade” that poses diagnostic and management challenges.

## Case presentation

A 54-year-old male patient was referred to our hospital due to progressive jaundice. His symptoms began in November 2024 with painless skin and scleral icterus, dark urine, and diarrhea (4–5 yellow, non-formed stools per day, related to food intake). The jaundice progressively worsened, and by January 2025, he developed pruritus and clay-colored stools.

Initial imaging examination at a local hospital in February 2025 revealed a left renal mass and a pancreatic head mass with multiple pancreatic cysts. Upon presentation to our institution, laboratory tests showed significant cholestasis: total bilirubin 137.2 μmol/L, direct bilirubin 113.8 μmol/L, GGT 655 U/L, ALP 295 U/L. Tumor marker CA19–9 was mildly elevated at 114 U/mL, while CA125, CA242, and CEA were within normal limits. This pattern raised suspicion for a periampullary or pancreatic head lesion causing biliary obstruction, but the mild CA19–9 elevation and absence of typical ductal adenocarcinoma markers suggested a possible neuroendocrine or other indolent etiology. Additional laboratory evaluation during follow-up showed mild elevations in pancreatic enzymes. On November 18, 2025, serum amylase was 180 U/L (reference range, 36–143 U/L) and serum lipase was 82 U/L (reference range, 2–53 U/L). Serum glucose was elevated at 9.4 mmol/L (reference range, 3.9–6.1 mmol/L). The most recent available lipid profile, obtained on May 6, 2025, showed total cholesterol of 5.87 mmol/L, triglycerides of 1.70 mmol/L, HDL cholesterol of 1.10 mmol/L, LDL cholesterol of 3.80 mmol/L, and non-HDL cholesterol of 4.77 mmol/L. Formal pancreatic exocrine function testing was not performed.

Contrast-enhanced computed tomography (CT) of the abdomen with pancreatic thin-slice sequencing revealed multiple cystic lesions of the pancreas with partial calcification, a solid-cystic mass in the pancreatic head with a solid component measuring 26×23 mm causing significant mass effect on the distal common bile duct, and a hyperenhancing nodule in the pancreatic neck (22×13 mm) suggestive of a neuroendocrine tumor (**Figure 1**). The hypervascular pancreatic lesion showed marked enhancement during the arterial phase, followed by decreased enhancement during the portal and delayed phases, an imaging pattern compatible with a pancreatic neuroendocrine tumor. Additionally, a large lobulated left renal mass (125×87 mm) highly suspicious for malignancy and multiple bilateral renal cysts, including a complex right renal cyst (Bosniak grade IV), were identified (**Figure 2**). The combination of pancreatic cysts, renal masses, and an early-onset retinal hemangioblastoma immediately suggested VHL syndrome as a diagnosis.

**Figure 1:**
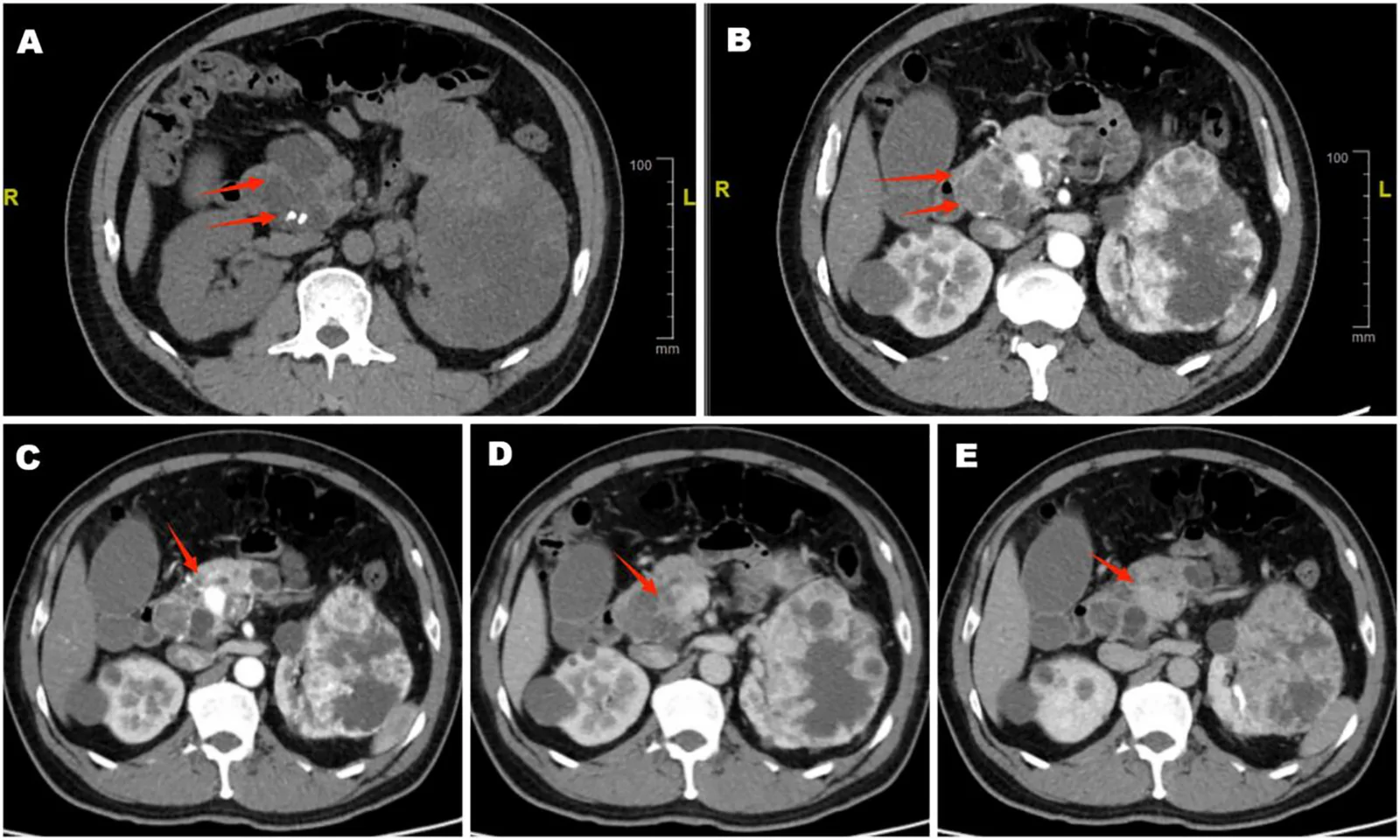
Contrast-enhanced CT imaging of the pancreas in the patient. **(A)** Multiple round hypodense lesions in the pancreas with calcification in some cyst walls. **(B)** A solid-cystic mass in the pancreatic head. The solid component shows mild enhancement on CT imaging, measuring approximately 26×23 mm on the largest axial section. **(C)** A hyperenhancing nodule in the pancreatic neck during the arterial phase. **(D, E)** Decreased enhancement in the portal and delayed phases. The nodule measures approximately 22×13 mm.

**Figure 2:**
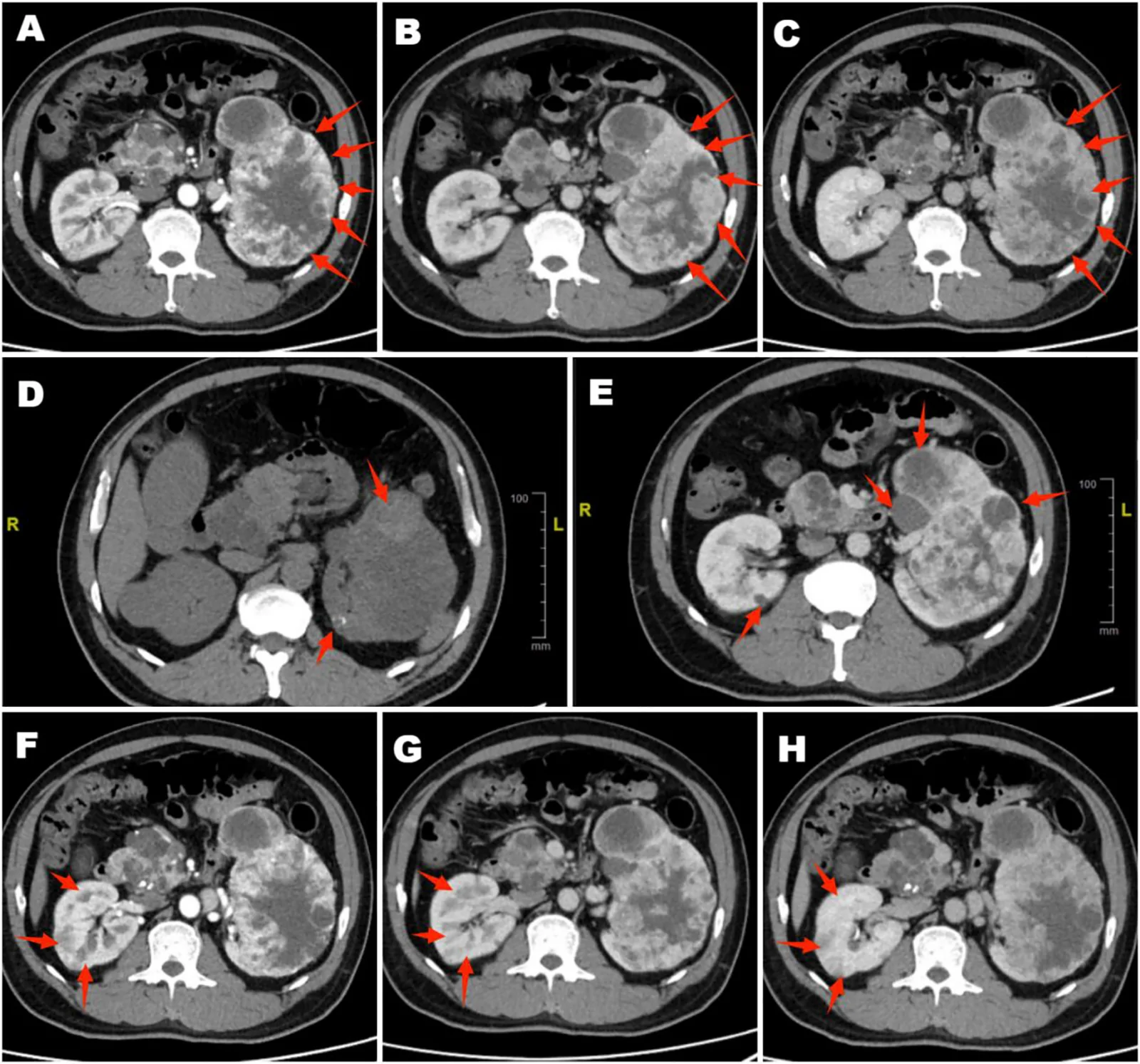
Contrast-enhanced CT imaging of the kidney in the patient. **(A-C)** A lobulated left renal mass with heterogeneous enhancement during the arterial phase **(A)** and marked peripheral enhancement. Enhancement decreases in the portal **(B)** and delayed phases **(C)**. The largest cross-section measures approximately 125×87 mm. The left upper and middle calyces are partially obscured by the mass. **(D-E)** Multiple round, non-enhancing hypodensities in both kidneys, some with calcification. The largest cyst measures approximately 24 mm in diameter. **(F-H)** A round, slightly hypodense lesion in the mid-portion of the right kidney with contrast enhancement, measuring approximately 13 mm in diameter.

Two different PET-CT examinations were subsequently performed for further characterization of the lesions. [^18^F]AlF-NOTA-LM3 somatostatin receptor PET-CT demonstrated increased tracer uptake in the 22×13 mm hyperenhancing pancreatic neck nodule (SUVmax 8.0), providing additional imaging support for an SSTR-positive neuroendocrine neoplasm (**Figure 3**). Separately, ^18^F-FDG PET-CT demonstrated increased metabolic activity in the left renal mass (SUVmax 16.5) (**Figure 4**), which was subsequently pathologically confirmed as ccRCC.

**Figure 3:**
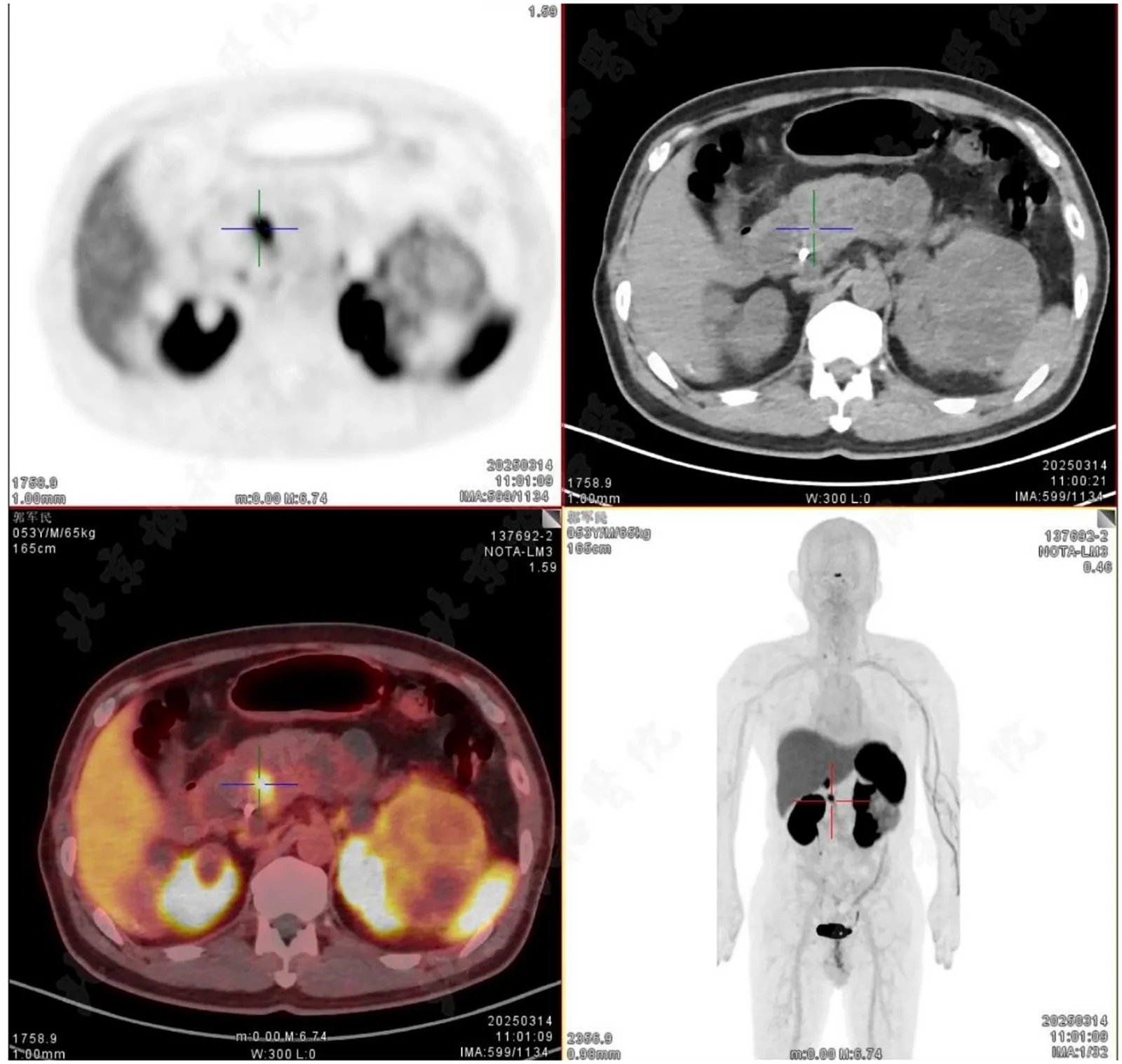
[^18^F]AlF-NOTA-LM3 somatostatin receptor PET-CT of the pancreatic neck nodule. Increased [^18^F]AlF-NOTA-LM3 uptake is observed in the 22 × 13 mm hyperenhancing pancreatic neck nodule (SUVmax 8.0), providing additional imaging support for an SSTR-positive neuroendocrine neoplasm.

**Figure 4:**
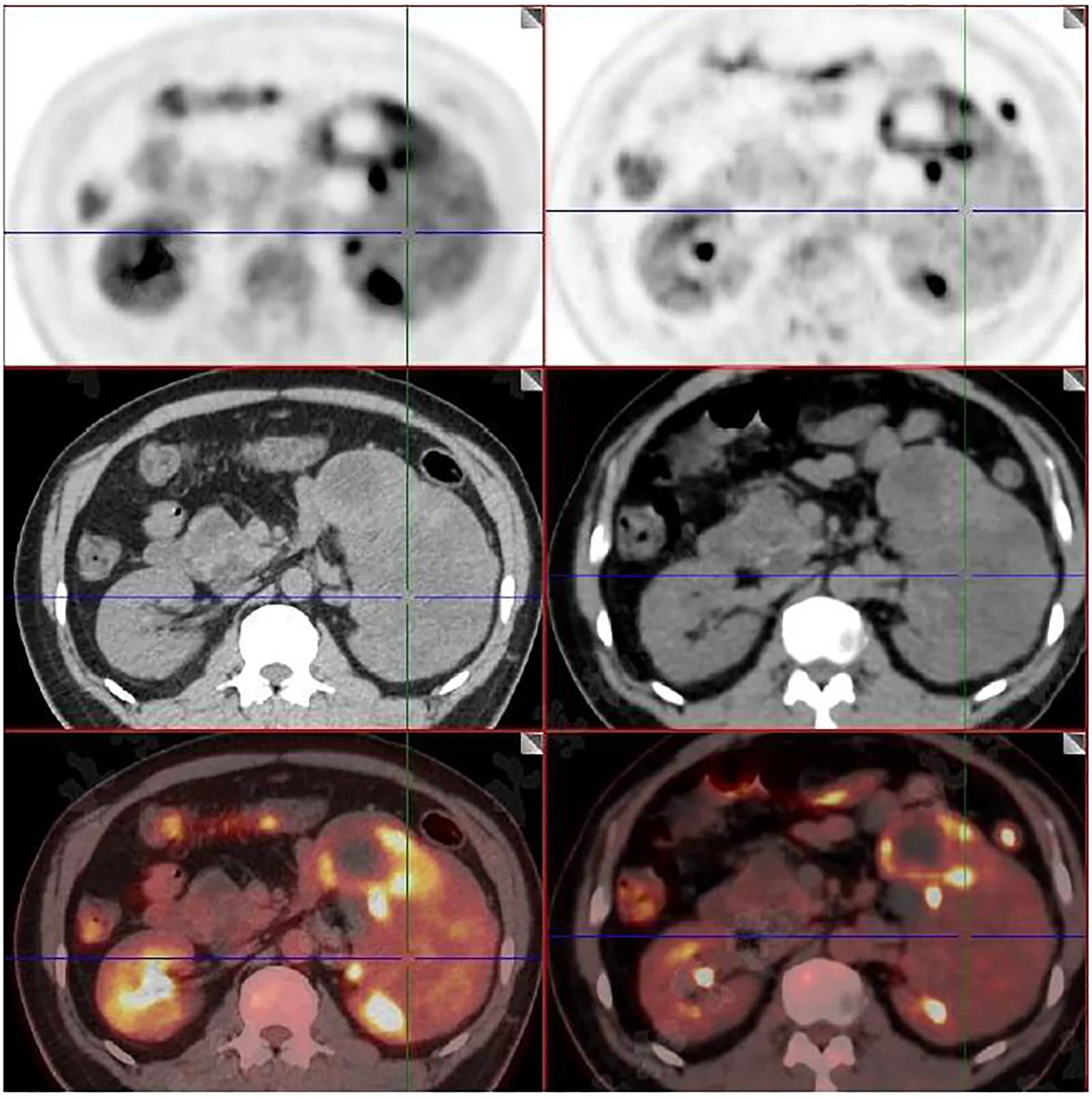
^18^F-FDG PET-CT shows a large hypermetabolic mass in the upper pole of the left kidney (SUVmax 16.5) and was subsequently pathologically confirmed as clear cell renal cell carcinoma. The apparent intense activity in the right kidney is localized to the renal pelvis/collecting system and represents physiological urinary excretion of ^18^F-FDG rather than abnormal renal parenchymal uptake.

Further endoscopic evaluation clarified the mechanism of biliary obstruction. As **Figure 5** shows, endoscopic ultrasound (EUS) demonstrated dilatation of the common bile duct to approximately 16.1 mm, multiple cystic lesions in the pancreatic head, and a hypoechoic solid lesion in the pancreatic head. Bile duct narrowing was observed at the level of this pancreatic head lesion. Endoscopic retrograde cholangiopancreatography (ERCP) subsequently demonstrated dilatation of the common bile duct with focal stenosis of its distal intrapancreatic segment. These findings were anatomically concordant with the pancreatic head cystic-solid lesion identified on contrast-enhanced CT. Because of progressive jaundice, the patient underwent biliary stent placement, which rapidly relieved the jaundice. Fine-needle aspiration (FNA) targeted the solid component of the pancreatic head cystic-solid lesion. The specimen yielded only a few atypical, highly degenerated cells, together with inflammatory exudate and small intestinal epithelial tissue, and was therefore non-diagnostic. Importantly, the separate 22×13 mm pancreatic neck nodule suspicious for pNET was not biopsied; therefore, its diagnosis remained imaging based. A subsequent ultrasound-guided percutaneous biopsy of the left renal mass was also non-diagnostic due to insufficient tissue. At this point, the clinical feature strongly pointed to VHL syndrome with possible pNET and ccRCC, but pathological confirmation was lacking.

**Figure 5:**
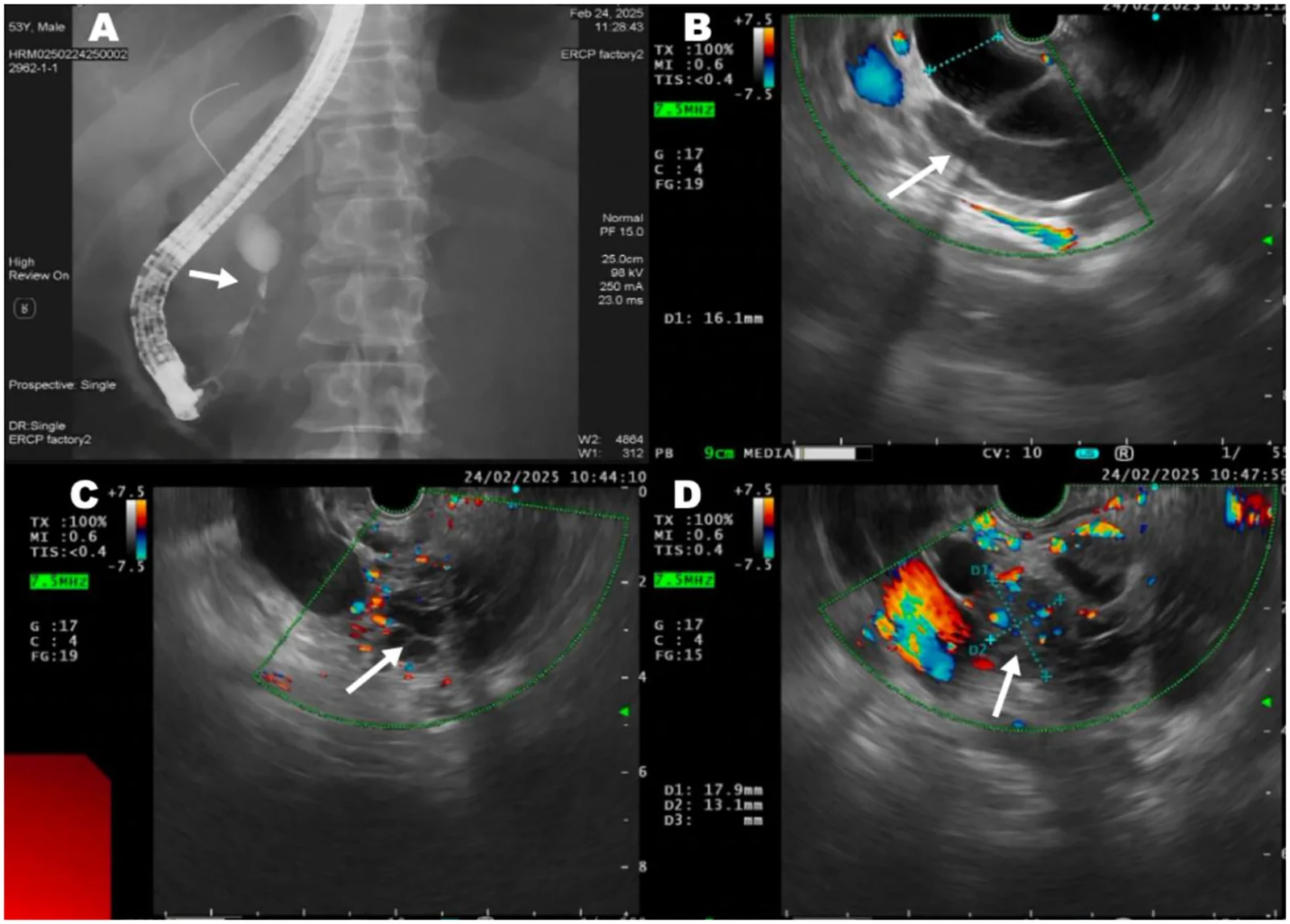
ERCP and EUS findings demonstrating the biliary obstruction. **(A)** ERCP shows dilatation of the common bile duct with focal stenosis of the distal intrapancreatic segment. **(B)** EUS demonstrates dilatation of the common bile duct to approximately 16.1 mm. **(C)** Multiple cystic lesions are present in the pancreatic head. **(D)** A hypoechoic solid lesion measuring approximately 17.9 × 13.1 mm is identified in the pancreatic head, with focal narrowing of the adjacent bile duct.

Given the large size and aggressive imaging features of the left renal mass, a multidisciplinary team recommended proceeding with left nephrectomy for both diagnostic and therapeutic purposes. Pathological examination confirmed a ccRCC (WHO grade 3) without invasion of the renal pelvis, capsule, or sinus fat, with negative margins. Immunohistochemistry showed positivity for PAX-8 (weak), AE1/AE3, CD10 (partial), EMA (weak), RCC, Vimentin, and CA9, while negative for CK7, TFE3, and CD117.

Over the same period, he reported an unintentional weight loss of 10 kg (from 78 kg to 68 kg). His past medical history was significant for a right retinal hemangioblastoma treated with cryotherapy in 1992, which eventually led to glaucoma, atrophy of eyeball, and replacement with a prosthetic eye in 1994. He also had a history of surgically resected rib tuberculosis in 1999 and hypertension managed with amlodipine. His family history is notable for a brother who died from an intracranial tumor at the age of 24. However, the exact pathological diagnosis was unavailable.

Physical examination on admission revealed scleral icterus, a prosthetic right eye, and a well-healed surgical scar on the left abdomen. The abdomen was soft and non-tender. Neurological examination was unremarkable.

During follow-up, germline genetic testing of peripheral blood using targeted capture next-generation sequencing (Illumina NextSeq 550DX) identified a VHL splice-site variant, NM_000551.3:c.464-2A>C, which was classified as likely pathogenic according to the ACMG 2015 criteria, confirming the molecular diagnosis of VHL disease. Cascade genetic screening was subsequently performed in the patient’s two sons. One son was found to carry the same VHL variant, whereas the other tested negative. The patient was phenotypically classified as having type 1 VHL disease because there was no clinical history of pheochromocytoma and no adrenal lesion suggestive of pheochromocytoma was identified on abdominal imaging. Because of the residual pancreatic lesion and the contralateral complex renal lesion, the patient initiated belzutifan at a dose of 120 mg/day in December 2025. Treatment was continued until March 2026 and was discontinued because of adverse events, including tinnitus, nausea, and nasal congestion. Detailed quantitative laboratory monitoring data during treatment were not available. In March 2026, follow-up contrast-enhanced CT performed at an outside hospital showed that the pancreatic and contralateral renal lesions remained essentially unchanged in size compared with the previous examination, with no radiographic evidence of disease progression. Exact comparative lesion measurements were not available from the outside-hospital report. The major clinical events and management course are summarized in **Table 1**.

**Table 1:** Timeline of VHL-related events.

Time point	Clinical events
1992	right retinal hemangioblastoma treated with cryotherapy
1994	glaucoma, atrophy of eyeball, and replacement with a prosthetic eye
2024-11	jaundice
2025-1	Development of pruritus and clay-colored stools
2025-2	examination revealed a left renal mass and a pancreatic head mass with multiple pancreatic cysts
2025-2	ERCP and biliary stent placement; pancreatic sampling
2025-2	^18^F-FDG PET-CT
2025-3	[^18^F]AlF-NOTA-LM3 PET-CT
2025-4	Left radical nephrectomy; ccRCC confirmed
2025-11	Admission; cholestatic liver tests and contrast-enhanced CT
2025-11	Germline VHL testing
2025-12	Belzutifan initiated
2026-2	Cascade testing of two sons
2026-3	Belzutifan discontinued; follow-up imaging showed short-term stability

## Discussion

We report a molecularly confirmed case of VHL disease phenotypically classified as type 1 characterized by extensive pancreatic and renal cystic involvement, a pathologically confirmed ccRCC and a pancreatic hypervascular lesion highly suspicious for pNET. Importantly, the pancreatic neck nodule suspected to represent pNET was not biopsied and therefore remained an imaging-based diagnosis; tissue sampling of the separate pancreatic head cystic-solid lesion was non-diagnostic. Furthermore, the available evidence does not establish that either the renal or pancreatic neoplasm arose from a pre-existing cyst. Rather, the principal clinical significance of this case lies in the coexistence of extensive cystic disease with solid neoplastic lesions, which complicated their recognition and characterization and created a “cystic masquerade”. The patient’s initial presentation with obstructive jaundice further posed substantial diagnostic challenges, highlighting the complexity of VHL-related pancreatic manifestations.

Pancreatic and renal cystic lesions are among the most common manifestations of VHL disease and are often considered relatively indolent. However, this perception may lead to underestimation of malignant potential. In our patient, multiple pancreatic cysts coexisted with a hypervascular pancreatic lesion and a cystic-solid mass in the pancreatic head, while bilateral renal cysts were accompanied by a giant ccRCC and a contralateral Bosniak IV cyst. These findings underscore that the presence of extensive cystic disease in VHL should not preclude careful evaluation for coexisting solid neoplastic lesions. Careful assessment of mural nodules, enhancing solid components, interval growth, and radiographic complexity is essential for identifying lesions requiring closer surveillance or intervention.

The differential diagnosis of pancreatic cystic lesions in VHL disease is broad and includes simple pancreatic cysts, serous cystadenomas, intraductal papillary mucinous neoplasms, mucinous cystic neoplasms, cystic degeneration of pNETs, and pancreatic ductal adenocarcinoma. Among these entities, the presence of hypervascular enhancement or an enhancing mural nodule should raise suspicion for pNET. In the present case, the lesion suspected to represent pNET was the separate 22×13 mm hyperenhancing nodule in the pancreatic neck. Suspicion of pNET was based on complementary multimodal imaging findings, including contrast-enhanced CT, findings on contrast-enhanced ultrasonography and EUS, and increased [^18^F]AlF-NOTA-LM3 uptake (SUVmax 8.0) on somatostatin receptor PET-CT. The molecular diagnosis of VHL disease further increased the clinical likelihood of a VHL-associated pNET. Regarding pancreatic function, mild elevations in serum amylase and lipase were observed during follow-up, together with an elevated serum glucose level. The patient’s diarrhea and weight loss also raised the possibility of impaired pancreatic exocrine function; however, these symptoms were nonspecific in the setting of biliary obstruction. Formal assessment of pancreatic exocrine function was not performed. Therefore, although the extensive pancreatic involvement may have affected pancreatic physiology, clinically significant pancreatic exocrine insufficiency could not be confirmed.

Of note, the patient presented with obstructive jaundice. VHL-associated pNETs are typically nonfunctional and asymptomatic, being detected incidentally during surveillance imaging. Obstructive jaundice is distinctly uncommon. Importantly, in this case, the biliary obstruction was not attributed to the separate pancreatic neck nodule suspected to represent pNET. ERCP demonstrated common bile duct dilatation with focal stenosis of the distal intrapancreatic common bile duct. EUS further showed a dilated common bile duct, multiple pancreatic head cystic lesions, and a hypoechoic solid lesion in the pancreatic head at the level of the biliary narrowing. Together with the anatomical relationship observed on contrast-enhanced CT, these findings suggest that local mass effect or extrinsic compression from the pancreatic head cystic-solid lesion was the most likely mechanism of biliary obstruction.

The diagnosis of VHL syndrome was substantially delayed. Retinal hemangioblastoma developed more than three decades before recognition of the underlying hereditary syndrome. During this interval, the patient developed a large ccRCC and multiple pancreatic lesions. This prolonged diagnostic delay emphasizes the importance of considering VHL disease in any patient presenting with early-onset retinal hemangioblastoma, central nervous system hemangioblastoma, or unexplained multisystem cystic lesions. Early recognition facilitates surveillance and allows intervention before the development of advanced organ involvement.

Contemporary management of gastrointestinal tumors increasingly emphasizes individualized treatment and organ preservation whenever oncologically appropriate (12). This principle is particularly relevant to VHL disease, in which patients may develop multiple bilateral or multifocal tumors over their lifetime. Given the indolent nature of many VHL-associated pNETs, a conservative approach with close imaging examination was adopted. Current guidelines recommend surgical intervention for VHL-related pNETs larger than 2–3 cm, those with rapid growth, or those that become symptomatic (11). In this patient, the lesion measured 2.2 cm, and further growth or development of symptoms may prompt reconsideration of surgical intervention. Also, the patient underwent radical left nephrectomy due to the large size of the renal mass and the strong suspicion of malignancy. Nevertheless, continued surveillance of the contralateral kidney is critical because VHL-associated renal tumors are frequently multifocal and bilateral. Partial nephrectomy remains the preferred approach when feasible to preserve renal function and avoid end-stage renal disease.

Targeted anticancer therapies are increasingly designed to improve treatment selectivity while limiting damage to normal tissues, an important principle of precision oncology (13). The recent FDA approval of belzutifan, a first-in-class HIF-2α inhibitor, has transformed the treatment landscape for VHL-associated RCC and other tumors (14, 15). For patients with confirmed germline VHL mutations, belzutifan offers a targeted approach that can reduce tumor burden while preserving organ function, particularly for renal and pancreatic lesions. In our patient, belzutifan was initiated at 120 mg/day in December 2025 and continued until March 2026. Treatment was discontinued because of adverse events, including tinnitus, nausea, and nasal congestion. Follow-up contrast-enhanced CT performed at an outside hospital in March 2026 showed no appreciable interval change in the residual pancreatic or contralateral renal lesions and no radiographic evidence of progression. Because detailed quantitative laboratory monitoring data and standardized comparative lesion measurements were unavailable, treatment response could not be formally quantified. Given the short duration of treatment and follow-up, the observed outcome should be interpreted as short-term radiographic disease stability rather than evidence of therapeutic efficacy. Longer-term follow-up is required to determine the durability of disease control.

VHL disease is inherited in an autosomal dominant manner with high penetrance. Although the patient’s brother had a history of an intracranial tumor, the diagnosis of VHL in this relative could not be confirmed. Importantly, germline testing identified a heterozygous VHL variant and subsequent family screening demonstrated that one of his two sons carried the same variant whereas the other tested negative. This finding highlights the importance of cascade genetic testing and genetic counseling in affected families. Early identification of variant carriers allows implementation of surveillance programs before the development of irreversible complications, including blindness from retinal hemangioblastoma, advanced renal cell carcinoma, and central nervous system hemangioblastomas.

This case provides an important clinical reminder that extensive cystic disease in VHL can complicate the recognition and characterization of coexisting clinically significant solid lesions. Recognition of suspicious imaging features within cystic lesions, including mural nodules, enhancing solid components, and interval growth, is critical for avoiding delayed diagnosis and optimizing patient outcomes. Early genetic evaluation, vigilant surveillance, and multidisciplinary management remain essential to improving long-term prognosis in patients with VHL syndrome.

### Strengths and limitations

Several strengths and limitations of this report should be acknowledged. The diagnosis of VHL disease was supported by the characteristic multisystem clinical phenotype and confirmed by germline genetic testing, while the renal malignancy was pathologically confirmed as ccRCC. Multimodal imaging, family cascade testing, and longitudinal clinical follow-up further strengthen the clinical characterization of this case. However, the pancreatic neck nodule suspected to represent pNET was not biopsied and therefore remains an imaging-based diagnosis. Furthermore, the available imaging and pathological evidence does not establish that either the renal or pancreatic neoplasm arose from a pre-existing cyst; therefore, the present case demonstrates coexistence rather than a direct cyst to tumor origin. In addition, the relatively short duration of belzutifan treatment and radiographic follow-up precludes any firm conclusions regarding therapeutic efficacy or long-term tumor control. Detailed quantitative laboratory monitoring data and standardized comparative measurements of the residual lesions during belzutifan treatment were also unavailable, further limiting formal assessment of treatment response and toxicity. Finally, as a single-patient case report, these observations cannot be generalized to the broader VHL population.

## Conclusion

To the best of our knowledge, this is a rare case of type 1 VHL syndrome presenting with obstructive jaundice and extensive pancreatic and renal cystic involvement coexisting with a pathologically confirmed ccRCC and an imaging-suspected pancreatic neuroendocrine tumor. Extensive cystic disease may complicate the recognition and characterization of coexisting solid neoplastic lesions and thereby contribute to diagnostic challenges. This report expands the phenotypic spectrum of VHL-associated pancreatic disease. Clinicians should maintain a high index of suspicion when evaluating pancreatic and renal cystic lesions in patients with suspected VHL disease, particularly when enhancing solid components or mural nodules are present. Early-onset retinal hemangioblastoma is a red flag requiring immediate VHL genetic testing, regardless of family history. Multidisciplinary management is essential to balance oncologic resection with organ preservation, and family screening must be initiated promptly upon diagnosis to prevent morbidity in undiagnosed relatives. Furthermore, emerging targeted therapies such as HIF-2α inhibitors offer new hope for these patients and should be considered in eligible individuals. In summary, this case enriches the clinical spectrum of VHL syndrome and highlights the importance of early genetic testing and multidisciplinary care.

## Data Availability

The original contributions presented in the study are included in the article/supplementary material. Further inquiries can be directed to the corresponding authors.

## References

[1] Latif F Tory K Gnarra J Yao M Duh FM Orcutt ML . Identification of the von Hippel-Lindau disease tumor suppressor gene. Science. (1993) 260:1317–20. doi: 10.1126/science.8493574 8493574

[2] Kim JJ Rini BI Hansel DE . Von hippel lindau syndrome. In: Ahmad SI , editor. Diseases of DNA Repair. Springer, New York, NY (2010). p. 228–49. 10.1007/978-1-4419-6448-9_22 20687511

[3] Melmon KL Rosen SW . Lindau’s disease. Review of the literature and study of a large kindred. Am J Med. (1964) 36:595–617. doi: 10.1016/0002-9343(64)90107-x 14142412

[4] Maher ER Neumann HP Richard S . Von Hippel-Lindau disease: a clinical and scientific review. Eur J Hum Genet EJHG. (2011) 19:617–23. doi: 10.1038/ejhg.2010.175 21386872 PMC3110036

[5] Gossage L Eisen T Maher ER . VHL, the story of a tumour suppressor gene. Nat Rev Cancer. (2015) 15:55–64. doi: 10.1038/nrc3844 25533676

[6] Gnarra JR Zhou S Merrill MJ Wagner JR Krumm A Papavassiliou E . Post-transcriptional regulation of vascular endothelial growth factor mRNA by the product of the VHL tumor suppressor gene. Proc Natl Acad Sci USA. (1996) 93:10589–94. doi: 10.1073/pnas.93.20.10589 8855222 PMC38197

[7] Iliopoulos O Levy AP Jiang C Kaelin WG Jr. Goldberg MA . Negative regulation of hypoxia-inducible genes by the von Hippel-Lindau protein. Proc Natl Acad Sci USA. (1996) 93:10595–99. doi: 10.1073/pnas.93.20.10595 8855223 PMC38198

[8] Lonser RR Glenn GM Walther M Chew EY Libutti SK Linehan WM . Von hippel-lindau disease. Lancet. (2003) 361:2059–67. doi: 10.1016/S0140-6736(03)13643-4 12814730

[9] Chen F Kishida T Yao M Hustad T Glavac D Dean M . Germline mutations in the von Hippel-Lindau disease tumor suppressor gene: correlations with phenotype. Hum Mutat. (1995) 5:66–75. doi: 10.1002/humu.1380050109 7728151

[10] Hoffman MA Ohh M Yang H Klco JM Ivan M Kaelin WG Jr. Von Hippel-Lindau protein mutants linked to type 2C VHL disease preserve the ability to downregulate HIF. Hum Mol Genet. (2001) 10:1019–27. doi: 10.1093/hmg/10.10.1019 11331612

[11] Keutgen XM Hammel P Choyke PL Libutti SK Jonasch E Kebebew E . Evaluation and management of pancreatic lesions in patients with von Hippel-Lindau disease. Nat Rev Clin Oncol. (2016) 13:537–49. doi: 10.1038/nrclinonc.2016.37 27030075

[12] Chen Q Yuan Y Linghu E . Advances in the diagnosis and treatment of gastrointestinal tumors under the concept of super minimally invasive surgery. J Transl Intern Med. (2025) 13:183–6. doi: 10.1515/jtim-2025-0023 40896286 PMC12392081

[13] Yan Y Zhang Y Liu J Chen B Wang Y . Emerging magic bullet: subcellular organelle-targeted cancer therapy. Med Rev. (2025) 5:117–38. doi: 10.1515/mr-2024-0044 40224364 PMC11987508

[14] Jonasch E Donskov F Iliopoulos O Rathmell WK Narayan VK Maughan BL . Belzutifan for renal cell carcinoma in von Hippel-Lindau disease. N Engl J Med. (2021) 385:2036–46. doi: 10.1056/NEJMoa2103425 34818478 PMC9275515

[15] Saad E Machaalani M McDermott DF Choueiri TK . The clinical landscape of HIF2α inhibitors in oncology. Nat Rev Clin Oncol. (2026) 23(7):501–19. doi: 10.1038/s41571-026-01145-y 42050152

